# The aspirational necessity of HIV prevention

**DOI:** 10.1002/jia2.25289

**Published:** 2019-05-17

**Authors:** Lawrence Corey, Glenda E Gray, Susan P Buchbinder

**Affiliations:** ^1^ Vaccine and Infectious Disease Division Fred Hutchinson Cancer Research Center Seattle WA USA; ^2^ Perinatal HIV Research Unit Faculty of Health Sciences University of the Witwatersrand Johannesburg South Africa; ^3^ Bridge HIV San Francisco Department of Public Health San Francisco CA USA

**Keywords:** HIV/AIDS, vaccine, end the epidemic/ETE, treatment as prevention/TASP, 90‐90‐90

While the rhetoric around the slogan “the ending of AIDS” rolls nicely off the podium, the reality of the situation on the ground is far from what even the most optimistic observers would call imminent. Globally, it is estimated there are 1.4 million new infections annually and in the US, progress has stalled at approximately 40,000 new infections yearly; a figure essentially unnoticed by the press or public because of the silent nature of HIV acquisition 1. While Treatment as Prevention (TASP) has been extolled as a means to control the HIV epidemic, the population‐based evidence has been tepid. The HPTN 071 study commonly known as PopART demonstrated a modest effect of universal test and treat in a community randomized trial in Zambia and South Africa, making this alone an unlikely tool to curtail the epidemic in South Africa. In addition, in more resource‐rich settings, TASP appears to be insufficient to fully control the HIV epidemic with evidence of a “re‐emerging epidemic” among young MSM and men who inject drugs 2, 3. Data from cities in the global north, like Vancouver, Canada, indicate that even with enhanced surveillance, and a concentrated public health response to rapid identification and treatment, HIV infections amongst men are still higher than the provincial average, with new HIV diagnoses among MSM in Vancouver increasing amongst younger men 4, 5. Mental health, substance use, homelessness, and stigma all contribute to challenges in reaching an entire population of persons at‐risk for or living with HIV. In other settings, one has to overcome structural barriers to access early care and ART adherence over time to stop the onward transmission of this infection. It is evident that we cannot simply treat our way out of this epidemic and it behooves us to develop the tools to improve our prevention strategies.

The history of sexually transmitted infections also tells us that infectious diseases that are acquired and transmitted asymptomatically require biomedical approaches to prevention; approaches that protect the person from acquiring infection when they least suspect their exposure. However, this too will require widespread roll‐out, as is required to prevent hepatitis B through vaccination, another sexually transmitted infection. If one wants to achieve what we would consider a goal of ending of the AIDS epidemic – a 90% reduction in current rates of new infections (<1000 new cases per year in the US or <150,000 cases globally) – we need to be innovative and require new tools and new commitments to develop such tools. This is the 90‐90‐90 goal that we in the prevention field should aspire to create: 90% reduction, 90% distribution and 90% effective coverage. We must develop strategies that will incrementally yet rapidly get us to these goals.

Development of a vaccine that achieves these targets is likely the best approach to controlling the HIV epidemic. Mathematical modelling suggests that even a partially effective vaccine will reduce forward transmission with less coverage, because of the difference in required adherence between repeated administration of an antibody or antiviral versus a longer acting vaccination. However, there are alternative biomedical approaches that could also offer major incremental benefits along the way. These include the administration of long‐acting broadly neutralizing antibodies that bind the virus to reduce its infectivity. This strategy can also be achieved by injection of a vector that then produces these antibodies persistently over time. Alternatively, a long‐acting injectable or implantable antiretroviral that provides effective concentrations of effective drugs is another approach under active investigation. Nevertheless, non‐vaccine approaches are likely more expensive both in cost of goods and the requirement for more frequent administrations than a vaccine. Clinical trials that objectively and reliably define reduction of transmission, durability and cost are needed to accurately predict which of these approaches alone or in combination can make a substantial impact on reducing infections in a region. We believe that the axiom “good products make good markets” applies to the field of HIV prevention. HIV is now the epidemic of three generations and we remain optimistic that the logistic and financial structures will be made available to bring such interventions to the most affected communities globally.

The development of an effective HIV vaccine has been a more difficult challenge than we all initially assumed. We as a scientific community have stuttered and stalled with different approaches both logistically and scientifically. We have learned a few certainties along the way: that HIV vaccine research requires long‐term commitment and sustainable collaborations are necessary. Clinical trials are critical, especially large‐scale efficacy trials, because despite the innovation and insights that animal models provide, none yet predict the effectiveness in humans. We have, in our opinion, finally started to achieve a harmonization in scientific cooperation that is bringing better clarity and faster speed of development than ever before. Conceptually, we have field trials to evaluate the role both neutralizing and non‐neutralizing antibodies play in reducing HIV acquisition. Importantly, the trials are done in collaboration with an international group of scientists and computational biologists that will teach us what immune responses are important or not important in influencing acquisition. These trials are designed not just to define effectiveness, but to provide immunological insights into how to improve and succeed with future experiments. It is this type of international scientific effort that provides the optimism to reach these goals. Success must be defined as guiding us forward from partial to high‐level efficacy; from complex to simpler regimens; from hard‐to‐distribute to easy‐to‐distribute. Scientific insights will lead to these incremental improvements, which must then be implemented. Even 60% effective vaccine regimens can save millions of lives and reduce the strain on health care systems burdened with ever increasing numbers of persons on antiretrovirals. The good must be implemented before we achieve the perfect.

We live in a fortunate time. The tools of science are almost limitless in their ability to innovate and discover. Thanks to support from the Bill & Melinda Gates Foundation and the National Institutes of Health, sustained commitment to HIV prevention continues. The quality of science in the field of HIV is at world class levels. We have no doubt we will achieve the type of 90‐90‐90 prevention goal outlined above.

## Competing interests

LC and GEG declare no competing interests. SB has been an investigator for which Gilead Sciences has donated study drug.

## Authors’ contributions

LC wrote the first draft and made substantial edits. SB and GEG provided substantive edits to the manuscript.
